# Disproportional ventilatory response to incremental exercise in individuals with cerebral palsy

**DOI:** 10.1111/dmcn.70164

**Published:** 2026-01-18

**Authors:** Linnéa Corell, Emma Hjalmarsson, Rodrigo Fernandez‐Gonzalo, Annika Kruse, Sebastian Edman, Asta Kizyte, Rouli Wang, Arnoud Edelman Bos, Peder Sörensson, Eva Pontén, Petra E. M. van Schie, Annemieke I. Buizer, Jessica Norrbom, Daniele A. Cardinale, Ferdinand von Walden

**Affiliations:** ^1^ Department of Women's and Children's Health, Division of Pediatric Neurology Karolinska Institutet Stockholm Sweden; ^2^ Medical Unit Allied Health Professionals, Women's and Children's Health, Karolinska University Hospital Stockholm Sweden; ^3^ Department of Laboratory Medicine, Division of Clinical Physiology Karolinska Institutet Stockholm Sweden; ^4^ Unit of Clinical Physiology, Karolinska University Hospital Stockholm Sweden; ^5^ Department of Human Movement Science, Sport and Health University of Graz Graz Austria; ^6^ KTH MoveAbility, Department of Engineering Mechanics Royal Institute of Technology, KTH Stockholm Sweden; ^7^ Amsterdam UMC Vrije Universiteit Amsterdam, Rehabilitation Medicine Amsterdam Netherlands; ^8^ Amsterdam Movement Sciences, Rehabilitation & Development Amsterdam Netherlands; ^9^ Department of Medicine Solna Karolinska Institutet Stockholm Sweden; ^10^ Department of Cardiology Karolinska Institutet Stockholm Sweden; ^11^ Department of Pediatric Orthopedic Surgery Karolinska University Hospital Stockholm Sweden; ^12^ Emma Children's Hospital, Amsterdam UMC Amsterdam Netherlands; ^13^ Department of Physiology and Pharmacology Karolinska Institutet Stockholm Sweden; ^14^ The Swedish Sports Confederation (Riksidrottsförbundet) Stockholm Sweden

## Abstract

**Aim:**

To explore the integrated cardiopulmonary, metabolic, and muscular response to incremental exercise in individuals with cerebral palsy (CP) compared with typically developing participants.

**Method:**

This was a prospective cross‐sectional study. Sixteen (seven male) individuals with CP (classified in Gross Motor Function Classification System levels II–V) and 30 (15 male) typically developing participants performed a treadmill‐based incremental submaximal test and an exercise test to task failure. Participants used running frames (CP) or performed traditional running (typically developing participants). Metabolic and cardiopulmonary parameters were measured during both tests. Electromyography of the vastus lateralis and gastrocnemius medialis was recorded during the test to task failure.

**Results:**

Compared with typically developing participants, individuals with CP showed decreased minute ventilation (*p* < 0.05), increased respiratory frequency at a comparable exercise intensity (*p* < 0.05), and an altered metabolic response, on the basis of the partial pressure of carbon dioxide (*p* < 0.05) and lactate levels (*p* < 0.001), during both tests. In addition, participants with CP exhibited a lower ventilatory efficiency during the test to task failure (*p* < 0.01). Electromyography analysis suggested peripheral skeletal muscle fatigue in the lower limbs (*p* < 0.05) in individuals with CP compared with typically developing participants.

**Interpretation:**

Individuals with CP have a disproportional ventilatory response to incremental exercise, not driven by metabolic perturbations. The increased breathing frequency resulted in high rate of perceived exertion and signs of peripheral muscle fatigue compared with typically developing participants. Our findings stress the importance of interventions focused on ventilatory function in individuals with CP.

## Graphical abstract

Individuals with cerebral palsy demonstrate a disproportionate ventilatory response during incremental exercise. Compared with typically developing peers, respiratory frequency increases earlier and at comparable exercise intensities, contributing to higher perceived exertion and reduced ventilatory efficiency. At task failure, respiratory frequency is similar between groups, whereas heart rate is lower in individuals with cerebral palsy, likely reflecting a lower achieved exercise intensity.

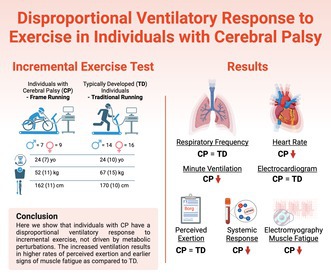

**Abbreviations**
FEV_1_
: forced expiratory volume in 1 second
FVC: forced vital capacity
*p*CO_2_
: partial pressure of carbon dioxide

**What this paper adds**

- Individuals with cerebral palsy (CP) show restrictive lung patterns during incremental exercise.
- Individuals with CP show a disproportionate elevated respiratory frequency during incremental exercise.
- Individuals with CP show a reduced ventilatory efficiency during high‐intensity exercise.
- Peripheral muscle fatigue is observed in individuals with CP during high‐intensity exercise.

Cerebral palsy (CP) is the most common childhood‐onset motor disorder, characterized by impairments in movement, posture, and coordination, originating from early insults to the developing brain.^1^, ^2^ Poor motor control predisposes for reduced physical activity, and a negative relationship between Gross Motor Function Classification System (GMFCS) levels and physical activity has been described, suggesting that individuals with more severe motor impairments engage the least in physical activity.^3^ Moreover, CP is associated with diminished cardiopulmonary fitness,^4^, ^5^ affecting both short‐term health and long‐term morbidity and mortality, predisposing individuals with CP to develop non‐communicable diseases including cardiovascular and respiratory disorders.^6^

During physical activity, pulmonary ventilation plays a central role in maintaining physiological homeostasis, with minute ventilation modulated by the interdependence of respiratory frequency and tidal volume. However, respiratory frequency and tidal volume are believed to be regulated by different inputs.^7^, ^8^ Respiratory frequency is primarily driven by non‐metabolic factors, including central command and muscle afferent feedback. In contrast, tidal volume is strongly regulated by metabolic stimuli, such as changes in blood pH and partial pressure of carbon dioxide (*p*CO_2_), mediated by peripheral and central chemoreceptors. Moreover, respiratory frequency and tidal volume differ in the timing of their responses. Respiratory frequency increases rapidly at the onset of exercise, while tidal volume responds more gradually. Together, they optimize ventilation to meet the demands of physical activity. Concomitantly, peripheral factors modulate the production of CO_2_ and are influenced by differences in skeletal muscle physiology related to bioenergetics. For example, differences in skeletal muscle fibre type between individuals, with ensuing differences in mitochondrial content and function, result in individual variations in oxidative capacity.^9^, ^10^ Likewise, the capillary density of skeletal muscle affects the extraction of oxygen during physical activity and a high capillary‐to‐fibre ratio is typically seen in skeletal muscle of endurance‐trained individuals.^11^, ^12^

Several of the aforementioned central and peripheral factors known to influence aerobic capacity are believed to be negatively affected in individuals with CP.^13^, ^14^, ^15^, ^16^ Recent studies indicate that individuals with CP have reduced respiratory function, characterized by low tidal volumes and reduced forced vital capacity (FVC).^17^, ^18^ In addition, individuals with CP often exhibit a high respiratory frequency during exercise compared with typically developing individuals,^5^, ^19^, ^20^ for reasons that are not yet fully understood. In our previous study, a significantly higher rate of perceived exertion at lower levels of exercise intensity in individuals with CP compared with typically developing individuals was observed.^21^ Although the level of perceived exertion is known to be correlated with heart rate, strong associations with respiratory frequency have also been observed,^22^ suggesting a high ventilatory effort in individuals with CP. This could indicate that, at a specific work intensity, the effort required for breathing may contribute more significantly to the perceived fatigue in individuals with CP than in typically developing participants.

Furthermore, individuals with CP demonstrate variations in muscle fibre type and architecture,^13^ alongside reduced mitochondrial abundance and function,^14^, ^15^ capillary density,^16^ and lower muscle mass.^13^ Collectively, these altered skeletal muscle characteristics can result in insufficient delivery of oxygen to peripheral working muscle, with increased lactate production as a consequence,^5^, ^23^ which may contribute to decreased aerobic capacity in individuals with CP.

To further understand exercise physiology in individuals with CP and to improve overall cardiopulmonary fitness, we performed a treadmill‐based incremental submaximal test and an exercise test to task failure. We hypothesized that individuals with CP breathe more frequently during endurance exercise because of a reduction in tidal volume and alterations in acid–base balance, as evidenced by decreased pH levels and elevated *p*CO_2_ levels. Together, these factors are expected to lead to increased ventilatory effort, which will negatively affect physical performance during exercise. To allow an integrative physiological analysis, including cardiopulmonary, metabolic, and skeletal muscle function, we assessed (1) cardiopulmonary breath‐by‐breath ventilatory gas data, (2) cardiac function including plasma biomarkers and electrocardiogram (ECG), (3) heart rate and perceived exertion, (4) repeated venous blood gases, and (5) skeletal muscle activity using surface electromyography (EMG).

## METHOD

### Study design

In this prospective cross‐sectional study, performed at the Swedish Sports Confederation Performance Laboratory, Bosön, Sweden, individuals with CP and typically developing participants performed two incremental treadmill‐based exercise tests: one submaximal test (in the result section referred to as the submaximal incremental treadmill test) and one test to task failure (in the result section referred to as the incremental exercise test to task failure), either by using running frames (participants with CP) or by performing traditional running (typically developing participants). Additionally, all participants underwent cardiopulmonary assessment. Written informed consent was obtained from all participants or caregivers for individuals younger than 18 years of age. All included experiments were approved by the Swedish Ethical Review Authority (2021–05116 and 2022–0665202).

### Participants and recruitment

Sixteen individuals with CP and 30 typically developing individuals, aged 13 to 40 years, were enrolled in this study. Age range was set wide to maximize potential recruitment of individuals with CP. All exercise tests were conducted in 2022, while cardiopulmonary assessment at rest was performed the following year. Inclusion criteria for individuals with CP were a confirmed diagnosis of CP, ability to comprehend and follow verbal instructions, and sufficient experience to use a running frame for at least 10 minutes. Recruitment of participants with CP was mainly performed through frame running clubs and social media. The typically developing individuals were recruited through word of mouth. All considered themselves healthy and not on any regular medication. Exclusion criteria for both groups included soft tissue or bony surgery during the previous 6 or 12 months respectively, or pain that could impact performance. The measurements of anthropometrics and characteristics of the participants are presented in Table 1. For individuals with CP, bodyweight was measured using a large electronic scale (Detecto 6550, Carterville, IL, USA), which facilitated weighing individuals seated in a wheelchair. Height was measured with a tape measure, with participants either lying on an examination table or standing, on the basis of their individual ability. When contractures were present, distances between anatomical landmarks were measured and summed up. The same methods were used to measure both the weight and height of typically developing participants. Additionally, all participants were asked to evaluate their level of physical activity using the Saltin‐Grimby Physical Activity Level Scale.^24^

**TABLE 1:** Characteristics of the study participants.

	Cerebral palsy	Typically developing
*n*	16	30
Sex (female/male)	9/7	15/14
Age (years:months)	23:0 (19:0–27:0)	23:6 (15:0–33:3)
Body mass (kg)	52.4 (10.9)	66.6 (15.0)*
Body height (cm)	161.8 (11.1)	170.2 (10.3)*
Body mass index (kg/m^2^)	19.9 (3.1)	22.8 (3.6)*
Haemoglobin (g/L)	147.8 (16.46)	141.5 (16.61)
GMFCS level (II/III/IV/V)	2/5/8/1	n/a
CP type (spastic/dyskinetic/ataxic)	8/7/1	n/a
Frame running experience (years:months)	7:10 (3:6)	n/a
SGPALS (1/2/3/4)	2/5/6/3	1/2/16/11

Data are presented as mean (standard deviation) for normally distributed variables, and as median (interquartile range) for non‐normally distributed variables.

Abbreviations: CP, cerebral palsy; GMFCS, Gross Motor Function Classification System; n/a, not applicable; SGPALS, Saltin‐Grimby Physical Activity Level Scale (20) (grading: 1, physically inactive; 2, some light physical activity; 3, regular physical activity and training; 4, regular hard physical training for competition sports).

* Significant difference between groups (*p* < 0.05).

### Cardiopulmonary characterization at rest

To assess cardiac electrical activity, an ECG (IntelliVue MX550 Patient Monitor, Philips Healthcare, Amsterdam, the Netherlands) was conducted in 14 participants with CP and 26 typically developing participants. The collected ECGs were assessed by a specialist in cardiology. As a measure of compromised contractile function, levels of amino (N)‐terminal pro‐brain natriuretic peptide were assessed in 14 participants with CP and 24 typically developing participants. The blood samples were analysed at the Karolinska University Laboratory, Stockholm, Sweden. A dynamic spirometry (COSMED Quark Cardio Pulmonary Exercise Testing, Rome, Italy), was performed to measure FVC and forced expiratory volume in 1 second (FEV_1_). Ten individuals with CP performed the test using a face mask (COSMED Face Mask, Rome, Italy), because of challenges in achieving a proper seal with a conventional mouthpiece, while 29 typically developing participants completed the test using a standard mouthpiece. Each participant completed at least three measurements. Peak values were selected for analysis if exhalation lasted at least 6 seconds or if a plateau was observed by the test leaders. Spirometry values were adjusted for age and height using the calculator at European Respiratory Society to obtain percentages from predicted values.^25^

### Incremental treadmill‐based exercise tests: submaximal and to task failure

All participants performed two incremental treadmill‐based exercise tests in the same session: (1) a submaximal test and (2) a test to task failure. The mean time between tests was 1 hour 51 minutes for individuals with CP and 1 hour 38 minutes for typically developing participants.

Individuals with CP used the running frame on a wide treadmill (Rodby v2, Vänge, Sweden), while typically developing individuals used a standard treadmill (Rodby RL 2000E, Sweden). For safety purposes, all participants wore a harness that was secured in the ceiling with a rope. In addition, individuals with CP had the running frame attached to the treadmill by a rope and were closely supervised by two members of the research staff. Cardiopulmonary parameters (heart rate and respiratory parameters) were recorded breath‐by‐breath and assessed in 10‐second intervals using a 30‐second rolling mean during both tests.^26^ The inclination at the start was set to 1% for all participants during both tests. Owing to differences in physical ability, the initial pace and subsequent increase in speed and/or inclination were individually adjusted on the basis of warm‐up and/or previous records on competitive frame running performance.

The submaximal test involved multiple 4‐minute intervals with 1‐minute rest in between. The test was complete when the lactate production exceeded clearance and/or when the test leader, on the basis of experience, judged that the participant had reached a high level of exertion.

Participants with CP started with an initial speed of 1.5 km/h to 7 km/h, while typically developing participants started at 6 km/h to 9 km/h. Both groups had the inclination set at 1% throughout the test. After each interval, the speed was increased by 0.5 km/h to 1 km/h for both groups. Baseline values were obtained from the first interval for each participant, and the two (CP *n* = 4; typically developing *n* = 7) or three (CP *n* = 10; typically developing *n* = 23) last steps, depending on the total number of intervals completed, were used to assess aerobic capacity. The third minute of each interval was analysed to assure a steady state of cardiopulmonary parameters.

The incremental exercise test to task failure was performed with continuous increments in speed every 30 seconds for typically developing participants (*n* = 30), starting at 6 km/h to 10 km/h, with increments of 0.5 km/h. Participants with CP (*n* = 14) followed individualized protocols in which speed and/or inclination was increased every 30 or 60 seconds. The starting speed ranged from 1.6 km/h to 6 km/h, with increments of 0.2 km/h to 0.5 km/h. For four participants with CP who were unable to run at higher speed but had not yet reached estimated peak cardiopulmonary performance, the incline was increased gradually up to 4% instead. Cardiopulmonary parameters were analysed in intervals of 5% of total test time to account for differences in test duration.

### Cardiopulmonary data collection during exercise

Perceived effort was assessed with the Borg Rating of Perceived Exertion scale. The Borg Rating was assessed after each interval of the submaximal test and at completion of the test to task failure. Participants were equipped with a heart rate monitor (SmartLAB, HRM, Heddesheim, Germany), and gas exchange was recorded via a face mask (COSMED Face Mask, Rome, Italy) connected to a gas analysis system (COSMED Quark Cardio Pulmonary Exercise Testing, Rome, Italy) with the corresponding software (Omnia Software, COSMED, Rome, Italy). Information on respiratory frequency (breaths/minute), tidal volume (L/min), minute ventilation (VE, L/min), respiratory exchange ratio (the ratio between metabolic production of CO_2_ and uptake of oxygen, VO_2_/VCO_2_), respiratory intensity ratio (tidal volume/FVC), and ventilatory efficiency (VE/VCO_2_) was processed.

Cardiovascular intensity was calculated by using 30‐second rolling mean heart rate divided by the maximal heart rate defined as HR_max_ = 220 − age (i.e. HR_mean_/HR_max_).^27^ Respiratory intensity ratio was determined as the ratio of tidal volume during exercise to FVC, measured during dynamic spirometry. Oxygen pulse was determined by dividing VO_2peak_ by HR_peak_. Breathing reserve, expressed as a percentage, was calculated as (MVV − VE_max_)/MVV × 100, where VE_max_ represents the maximal minute ventilation achieved during exercise and MVV the maximal voluntary ventilation. Maximal voluntary ventilation was estimated by multiplying FEV_1_, measured during dynamic spirometry, by 40. Lactate and *p*CO_2_ levels are expressed as the fold change from baseline levels for each group when analysing differences during both incremental tests. The criteria used for a maximum physiological response (at least three of five composite variables: heart rate, respiratory exchange ratio, lactate, Borg Rating of Perceived Exertion scale, signs of perceived exertion) and individual data for each participant are described in Table S1.

### Blood sampling and blood analysis during exercise

Topical anaesthetic cream (EMLA, Aspen Nordic, Ballerup, Denmark) was applied to the skin 1 hour before initial blood sampling to minimize pain and discomfort in adolescents and individuals with CP. Venous blood gases were obtained through a peripheral venous catheter, inserted into a vein in the fossa cubitalis of each participant. A venous blood gas was collected at rest as baseline. During the submaximal incremental test, blood was collected between each 4‐minute interval. For the incremental exercise test to task failure, blood was collected immediately after and 3 minutes after exercise. The blood samples were analysed for acid–base balance and lactate levels using a blood gas analyser (ABL800 Flex, Radiometer, Copenhagen, Denmark). If no peripheral venous catheter could be used (CP *n* = 2; typically developing *n* = 1), capillary blood was used to analyse lactate levels by a lactate analyser (Biosen C‐line, EKF Diagnostics, Barleben, Germany).

### Assessment of muscle fatigue by electromyography

Bipolar surface EMG signals of the vastus lateralis and gastrocnemius medialis were measured with wireless EMG sensors (Myon Aktos, sampling frequency 1 kHz) (CP = 10; typically developing = 17) during the incremental exercise test to task failure. EMG signals were recorded from both legs in individuals with CP (less affected leg vs most affected leg, as assessed by a physiotherapist). For typically developing participants, the investigated leg was randomized. The skin was shaved and cleaned with an alcohol wipe before placing the electrodes according to the surface EMG for a non‐invasive assessment of muscles (SENIAM) recommendations.^28^ The recorded EMG signals were filtered with bandpass (cutoff frequencies 20–450 Hz) and notch (50 Hz) filters. The filtered signal was visually inspected for motion artefacts and any remaining artefacts (the identified noisy samples ±250 adjacent samples) were removed from the time series. The preprocessed data were further segmented into periods of inactivity and muscle activation. The inactivity periods were removed from further analysis.

The EMG signals were evaluated for muscle fatigue by extracting the first principal component of the feature set *F*_PCA_ = (average rectified value, zero crossing, slope sign changes, mean frequency, median frequency, bandwidth of the EMG signal).^29^ The frequency domain features (mean frequency, median frequency, and bandwidth of the EMG signal) were estimated using the fast Fourier transform. The first principal component was then normalized to the difference between its minimum and maximum values of the whole trial and further referred to as the EMG Fatigue Index. Given the feature set, a downward trend of the EMG Fatigue Index values is expected if the muscle is experiencing fatigue.

### Statistical analysis

Normal distribution of data was assessed using a Shapiro–Wilk test. Descriptive data between groups were compared using an independent *t*‐test or Mann–Whitney *U* test. A two‐way mixed analysis of variance, or a mixed‐effects analysis in cases where data were not normally distributed, was used with time (within‐subject) and group (cerebral palsy vs typically developing, between‐subject) as factors. This approach was applied to compare changes in the EMG fatigue index during the incremental exercise test to task failure, as well as mean levels of cardiopulmonary and blood gas parameters during both exercise tests, including heart rate, respiratory frequency, tidal volume, minute ventilation, respiratory intensity ratio, adjusted respiratory frequency, respiratory exchange ratio, ventilatory efficiency, Borg Rating of Perceived Exertion, pCO₂, lactate, and pH. If an interaction (time vs group) was observed, multiple comparisons with Bonferroni correction were performed to identify where differences occurred. Statistical summary tables are presented in Table S2. Participants' characteristics and data from the cardiopulmonary characterization at rest are presented as means and standard deviations for normally distributed data, and as medians with interquartile range for non‐normally distributed data. Parameters related to the submaximal exercise test are presented as means and standard deviations, whereas parameters related to the maximal exercise test are presented as medians and interquartile range. The level of statistical significance was set to *p* < 0.05. Statistical analysis was performed using GraphPad Prism (version 10.4.1 for Mac, GraphPad Software, Boston, MA, USA).

## RESULTS

All participants, except for two individuals with CP, fulfilled at least three out of five physiological criteria indicative of maximal performance during the incremental exercise test to task failure. One of the participants who did not meet at least three criteria was excluded from the analysis of both exercise tests because of difficulties running on a treadmill. The data from the second individual were excluded solely from the analysis of the exercise test to task failure. Additionally, one participant with CP declined the face mask and the blood samples and was therefore excluded from all exercise test results. A detailed table of each participant's physiological response during the incremental exercise test to task failure is presented in Table S1 and raw data are available at (https://doi.org/10.6084/m9.figshare.30998299).

### Cardiopulmonary characterization at rest

Assessment with ECG showed no pathology in either group and analysis of levels of N‐terminal pro‐brain natriuretic peptide revealed no difference between individuals with CP (33.5 ng/L [26.0–51.8]) and typically developing (27.5 ng/L [20.8–41.8]) participants (*p* > 0.05). Values below detection level (<11 ng/L) were excluded (CP = 4, typically developing = 3). However, individuals with CP had 53% lower FVC (*p* < 0.001) and 51% lower FEV_1_ (*p* < 0.001) than typically developing participants. A similar reduction was observed when individuals with CP were compared with their own predicted values (Table 2). No signs of airway obstruction were observed, as indicated by a non‐significant difference between groups for FEV_1_/FVC.

**TABLE 2:** Dynamic spirometry in individuals with cerebral palsy and typically developing participants.

	Absolute values (L)		Predicted values (%)	
	Cerebral palsy	Typically developing	Cerebral palsy	Typically developing
FVC	2.3 (1.3)	4.9 (1.3)*	52.7 (28.3–82.0)	106.4 (99.0–122.0)*
FEV_1_	1.9 (1.0)	3.9 (1.0)*	57.2 (32.6)	104.8 (15.2)*
FEV_1_/FVC%	84.6 (14.7)	80.7 (8.7)	100.6 (26.8)	94.6 (9.0)

Data are presented as mean (standard deviation) for normally distributed variables, and as median (interquartile range) for non‐normally distributed variables.

Abbreviations: FEV_1_, forced expiratory volume in 1 second; FVC, forced vital capacity.

* Significant difference between groups (*p* < 0.05).

### Cardiopulmonary response to the submaximal incremental treadmill test

**FIGURE 1:**
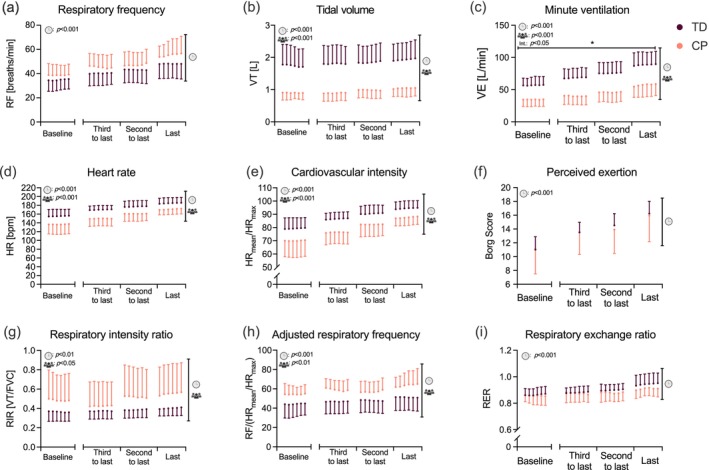
(a) Respiratory frequency, (b) tidal volume, (c) minute ventilation, (d) heart rate, (e) cardiovascular intensity, (f) perceived exertion, (g) respiratory intensity ratio, (h) adjusted respiratory frequency, and (i) respiratory exchange ratio during the submaximal incremental test in individuals with cerebral palsy and typically developing individuals. The *x*‐axis represents four steps: baseline, reflecting measurements during first step; third to last, corresponding to the third to last completed step; second to last, representing penultimate step; last, final step of the test. Values are presented as mean (standard deviation); 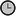 main effect of time (*p* < 0.05); 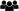 main effect of group (*p* < 0.05). If a significant interaction was observed, post hoc analysis was performed to identify differences between groups at specific time point, with significant effects (*p* < 0.05) denoted by an asterisk. Abbreviations: CP, cerebral palsy; FVC, forced vital capacity; HR, heart rate; Int., interaction; RER, respiratory exchange ratio; RF, respiratory frequency; RIR, respiratory intensity ratio; TD, typically developing; VE, minute ventilation; VT, tidal volume.

We first explored the cardiopulmonary response to a submaximal incremental exercise test in individuals with CP and typically developing participants. Data collected during the first interval (baseline) were compared with the three last intervals for each participant. Starting with respiratory parameters, we observed a main effect of time (*p* < 0.001, *F* = 86.63), indicating increased respiratory frequency over time in both groups (Figure 1a). However, contrary to our hypothesis, no interaction (time vs group) or main effect of group was observed. Assessment of tidal volume showed an increase over time (*p* < 0.001, *F* = 10.36) and group (*p* < 0.001, *F* = 32.92), where individuals with CP exhibited lower tidal volumes compared with typically developing participants (Figure 1b). This translated into a different response in minute ventilation in individuals with CP, where an interaction was observed (*p* = 0.026, *F* = 3.2) (Figure 1c). Collectively, these data suggest lower tidal volumes and lower minute ventilation during submaximal exertion in individuals with CP.

As a measure of exercise intensity, heart rate was evaluated throughout the submaximal test (Figure 1d). Main effects were observed for time (*p* < 0.001, *F* = 99.98) and group (*p* < 0.001, *F* = 13.96), driven by an increase in heart rate as intensity increased, and a lower heart rate in individuals with CP, suggesting that the submaximal exercise intensity in participants with CP was lower than in typically developing participants. The findings for heart rate persisted when evaluating cardiovascular intensity (HR_mean_/HR_max_), with main effects of time (*p* < 0.001, *F* = 102.1) and group (*p* < 0.001, *F* = 13.47) (Figure 1e).

Even though individuals with CP worked at a lower intensity, both groups experienced increased levels of perceived exertion (Figure 1f) throughout the test with a main effect of time (*p* < 0.001, *F* = 154.0).

The respiratory intensity ratio (tidal volume/FVC) showed a main effect of time (*p* = 0.003, *F* = 10.45) and group (*p* = 0.031, *F* = 5.05), indicating that individuals with CP used a larger portion of their FVC during exercise compared with typically developing individuals (Figure 1g).

Respiratory frequency adjusted for cardiovascular intensity revealed a main effect of time (*p* < 0.001, *F* = 26.39) and group (*p* = 0.008, *F* = 7.72), driven by higher overall values in individuals with CP (Figure 1h).

The assessment of respiratory exchange ratio (Figure 1i) showed a main effect of time (*p* < 0.001, *F* = 24.53), suggesting similar shifts in substrate utilization with increasing effort in both groups. Furthermore, an interaction was identified for ventilatory efficiency (*p* = 0.034, *F* = 2.99) (Figure S1), driven by higher ratios in individuals with CP throughout the submaximal incremental test.

### Systemic response to the submaximal incremental treadmill test

**FIGURE 2:**
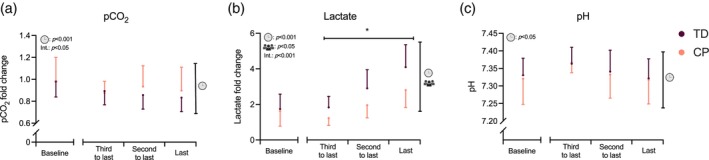
Blood gas analysis of (a) normalized *p*CO_2_, (b) normalized lactate, and (c) pH during the submaximal incremental test in individuals with cerebral palsy and typically developing individuals. The *x*‐axis represents four steps: baseline, reflecting measurements during first step; third to last, corresponding to the third to last completed step; second to last, representing penultimate step; last, final step of the test. Values are presented as mean (standard deviation); 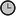 main effect of time (*p* < 0.05); 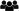 main effect of group (*p* < 0.05). If a significant interaction was observed, post hoc analysis was performed to identify differences between groups at a specific time point, with significant effects (*p* < 0.05) denoted by an asterisk. Abbreviations: CP, cerebral palsy; Int., interaction; *p*CO_2_, partial pressure of carbon dioxide; TD, typically developing.

Given the observed disproportional ventilatory response (increased respiratory frequency/cardiovascular intensity) to submaximal exercise in individuals with CP, we analysed venous blood gases during the exercise test to assess acid–base balance, lactic acid, and *p*CO_2_ levels. Contrary to our hypothesis, the analysis showed an interaction (time vs group) in normalized *p*CO_2_ (*p* = 0.031, *F* = 3.070) (Figure 2a). Similarly, lactate levels (*p* < 0.001, *F* = 9.564) (Figure 2b) displayed a greater response to exercise in the typically developing participants at the three last steps of the test. For pH, a general effect of time (*p* = 0.015, *F* = 4.362) was identified (Figure 2c).

### Cardiopulmonary response to the incremental exercise test to task failure

**FIGURE 3:**
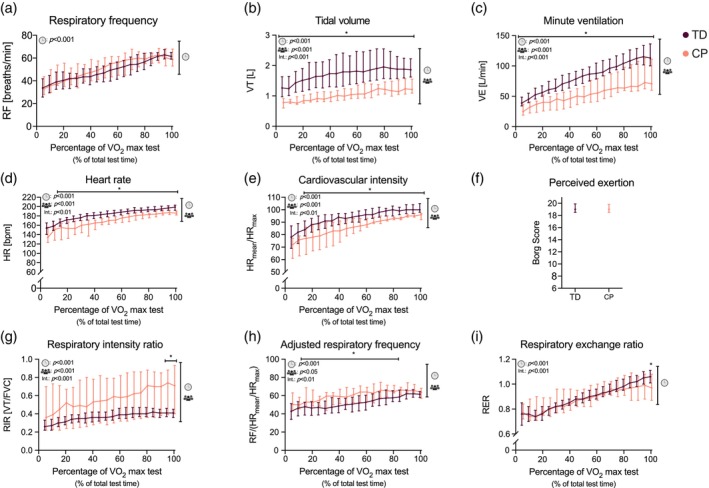
(a) Respiratory frequency, (b) tidal volume, (c) minute ventilation, (d) heart rate, (e) cardiovascular intensity, (f) perceived exertion, (g) respiratory intensity ratio, (h) adjusted respiratory frequency, and (i) respiratory exchange ratio during the incremental exercise test to task failure in individuals with cerebral palsy and typically developing individuals. The *x*‐axis represents the percentage of total test time. Values are presented as median (interquartile range); 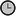 main effect of time (*p* < 0.05); 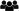 main effect of group (*p* < 0.05). If a significant interaction was observed, post hoc analysis was performed to identify differences between groups at specific time point, with significant effects (*p* < 0.05) denoted by an asterisk. Abbreviations: CP, cerebral palsy; FVC, forced vital capacity; HR, heart rate; Int., interaction; RER, respiratory exchange ratio; RF, respiratory frequency; RIR, respiratory intensity ratio; TD, typically developing; VE, minute ventilation; VO_2_, oxygen uptake; VT, tidal volume.

An incremental exercise test to task failure was conducted to further explore the cardiopulmonary response to high‐intensity exercise in individuals with CP. For respiratory frequency, there was a main effect of time (*p* < 0.001) (*F* = 99.72), driven by a general increase in respiratory frequency over time in both groups (Figure 3a). In concordance with the submaximal incremental test, no interaction (time vs group) or main effect of group was identified, suggesting that both groups increased their respiratory frequency at the same rate. An interaction was observed for tidal volume (*p* < 0.001) (*F* = 2.62) (Figure 3b), translating into an interaction in minute ventilation (*p* < 0.001, *F* = 4.27) (Figure 3c). This is indicative of an altered response throughout the test, driven by smaller tidal volume in individuals with CP compared with typically developing participants.

Heart rate showed an interaction (*p* = 0.003, *F* = 2.15) (Figure 3d), as did cardiovascular intensity (*p* = 0.001, *F* = 2.30) (Figure 3e), suggesting lower exercise intensity among individuals with CP.

All participants rated a comparable level of exertion at completion of the test (*p* = 0.63) (Figure 3f).

There was an interaction for respiratory intensity ratio (*p* < 0.001, *F* = 9.98), indicating higher respiratory effort in individuals with CP, as previously seen during the submaximal incremental test (Figure 3g). Further, respiratory frequency adjusted for cardiovascular intensity revealed an interaction (*p* = 0.004, *F* = 2.12) (Figure 3h), where individuals with CP showed generally higher values than typically developing individuals. An interaction for respiratory exchange ratio was observed (*p* < 0.001, *F* = 4.49), suggesting group‐level differences in substrate utilization, potentially reflecting variations in aerobic and anaerobic energy production (Figure 3i).

### Systemic response to the incremental exercise test to task failure

**FIGURE 4:**
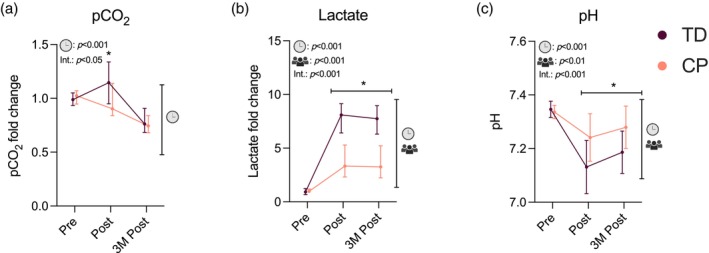
Blood gas analysis of (a) normalized *p*CO_2_, (b) normalized lactate, and (c) pH during the submaximal incremental test in individuals with cerebral palsy and typically developing individuals. The *x*‐axis represents three time points: Pre, values at rest; Post, immediately after exercise; 3M Post, 3 minutes after exercise. Values are presented as median (interquartile range); 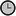 main effect of time (*p* < 0.05); 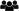 main effect of group (*p* < 0.05). If a significant interaction was observed, post hoc analysis was performed to identify differences between groups at a specific time point, with significant effects (*p* < 0.05) denoted by an asterisk. Abbreviations: CP, cerebral palsy; Int., interaction; *p*CO_2_, partial pressure of carbon dioxide; TD, typically developing.

There were interactions (time vs group) for both normalized *p*CO_2_ (*p* = 0.006, *F* = 5.471) and lactate levels (*p* < 0.001, *F* = 29.57), as well as for absolute pH (*p* < 0.001, *F* = 12.77) pre‐, post‐, and 3 minutes postexercise, indicative of milder systemic, metabolic response during high‐intensity exercise in individuals with CP compared with typically developing individuals (Figure 4).

### Cardiopulmonary limitations during the incremental exercise test to task failure

**FIGURE 5:**
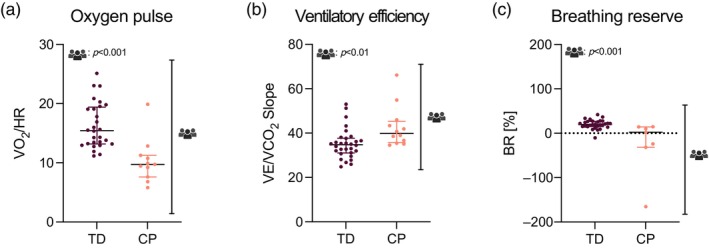
(a) Oxygen pulse, (b) ventilatory efficiency, and (c) breathing reserve for identification of cardiopulmonary limitations during the incremental exercise test to task failure in individuals with cerebral palsy (CP) and typically developing (TD) individuals. Values are presented on an individual basis with the line in the middle representing the median (interquartile range); 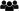 significant group difference (*p* < 0.05). Abbreviations: BR, breathing reserve; CP, cerebral palsy; HR, heart rate; TD, typically developing; VCO_2_, carbon dioxide production; VO_2_, oxygen uptake; VE/VCO_2_, ventilatory efficiency.

Individuals with CP showed a lower oxygen pulse compared with typically developing individuals (*p* < 0.001) during the exercise test to task failure (Figure 5a), indicating reduced oxygen consumption per heartbeat. The slope analysis of ventilatory efficiency revealed a steeper slope in individuals with CP (*p* = 0.002) (Figure 5b), strengthening the observation of reduced ventilatory efficiency previously seen during the submaximal incremental exercise test. Breathing reserve at task failure in individuals with CP was lower than for typically developing participants (*p* < 0.001), suggesting a higher utilization of maximal lung capacity in individuals with CP (Figure 5c).

### Muscle fatigue assessed by EMG during the incremental exercise test to task failure

**FIGURE 6:**
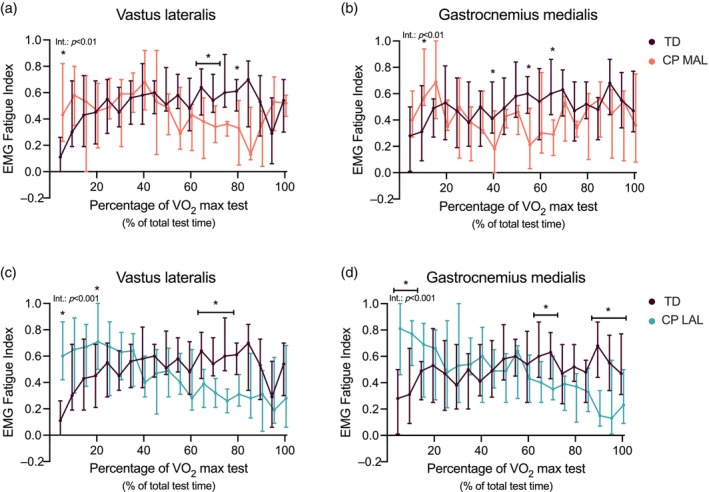
Electromyography of (a) vastus lateralis of the most affected leg, (b) gastrocnemius medialis of the most affected leg, (c) vastus lateralis of the less affected leg, and (d) gastrocnemius medialis of the less affected leg during the incremental exercise test to task failure in individuals with cerebral palsy and typically developing individuals. Values are presented as median (interquartile range). As significant interactions were observed for all analysis, post hoc analysis was performed to identify differences between groups at a specific time point, with significant effects (*p* < 0.05) denoted by an asterisk. Abbreviations: CP, cerebral palsy; EMG, electromyography; Int., interaction; LAL, less affected leg; MAL, most affected leg; TD, typically developing; VO_2_, oxygen uptake.

The analysis of peripheral fatigue by the EMG Fatigue Index in the lower extremities showed interactions (time vs group) for the vastus lateralis muscle in both the most affected leg (*p* = 0.004, *F* = 2.12) (Figure 6a) and the less affected leg (*p* < 0.001, *F* = 4.08) (Figure 6c) compared with typically developing participants. For the gastrocnemius medialis muscle, significant interactions (time vs group) were also observed for the most affected leg (*p* = 0.01, *F* = 1.95) (Figure 6b) and the less affected leg (*p* < 0.001, *F* = 3.86) (Figure 6d). Furthermore, individuals with CP experienced a negative drop in the EMG Fatigue Index throughout the test in both limbs compared with typically developing individuals. Collectively, this may suggest peripheral muscle fatigue in individuals with CP, which was not observed in typically developing participants.

## DISCUSSION

This is the first study, to our knowledge, exploring the integrated cardiopulmonary, metabolic, and skeletal muscle response to incremental submaximal exercise and exercise to task failure in individuals with CP. Our findings suggest that individuals with CP have an altered metabolic response and a disproportionately high respiratory frequency at a comparable exercise intensity than typically developing individuals. This breathing pattern is concomitant with evidence of peripheral muscle and exertional fatigue. Furthermore, consistent with previous findings,^17^, ^30^ individuals with CP demonstrated significantly lower lung volumes at rest and during exercise, providing further evidence of impaired ventilatory function potentially influencing exercise capacity.

Individuals with CP exhibited higher respiratory frequency than typically developing participants throughout both incremental tests when adjusted for cardiovascular intensity. These findings are consistent with our previous observations of high respiratory frequency during the 6‐Minute Frame Running Test among participants with CP.^19^ Interestingly, despite lower levels of metabolic indicators of exertion such as *p*CO_2_ and lactate levels in individuals with CP, respiratory frequency was markedly elevated. This supports the notion that respiratory frequency in individuals with CP is probably driven by behavioural components, including central command or a coping strategy to achieve greater minute ventilation, rather than by metabolic stimuli.^7^, ^8^ The observed pattern might also reflect impaired ventilatory control mechanisms or altered respiratory muscle function, tentatively contributing to the inefficiency in ventilation observed in this population.

Furthermore, individuals with CP demonstrated significantly lower lung volumes at rest and during exercise than typically developing participants, raising questions about the underlying contributing mechanisms. One interpretation of these data is that individuals with CP have a restrictive lung function pattern given the low FVC and FEV_1_, but an unaltered FEV_1_/FVC ratio measured during dynamic spirometry in this study.^31^ Alternatively, it could be attributed to ineffective coordination, increased muscle tone, and/or muscle weakness of respiratory muscles during breathing, factors influenced by the neurological origin of the condition. The chest plate of the running frame used by individuals with CP might also act as a limiting factor, potentially restricting lung expansion during activity. However, as the same phenomenon was observed at rest in individuals with CP, the contribution of the chest plate is probably minimal. Regardless, our findings suggest that high breathing frequency, rather than cardiovascular intensity, might be the primary driver of perceived exertion in individuals with CP, a finding consistent with observations made in typically developing individuals by Nicolo et al.^22^

To assess whether individuals with CP experience cardiovascular, gas exchange, and/or ventilatory limitations, we evaluated oxygen pulse, ventilatory efficiency, and breathing reserve under the exercise test to task failure, as these measures are recommended to be reported in standard clinical cardiopulmonary exercise testing.^32^ Our findings showed that individuals with CP had a lower oxygen pulse compared with typically developing participants, suggesting respiratory issues, reduced stroke volume, and/or peripheral oxygen extraction during exercise. Our data align with previous studies by Verschuren et al.^4^ and Balemans et al.,^5^ also reporting decreased oxygen pulse in participants with CP compared with typically developing participants. As stroke volume is believed to be similar between individuals with CP and typically developing individuals,^33^ a negative influence of the restrictive breathing pattern and/or local muscle factors (e.g. decreased capillarization, altered mitochondrial function) on oxygen extraction seems plausible. Importantly, in this study, the lower oxygen pulse is unlikely to be influenced by haemoglobin values, as there were no differences between the groups.

Furthermore, individuals with CP demonstrated altered ventilatory efficiency, as evidenced by an increased ratio in ventilatory efficiency during submaximal incremental exercise and a steeper slope during the test to task failure, similar to previous reports.^20^ Similar decrements in ventilatory function have been associated with mortality in patients with cardiopulmonary disease, emphasizing the clinical significance of these data.^34^, ^35^, ^36^

Our data also revealed a reduced breathing reserve in individuals with CP, suggesting a greater reliance on their ventilatory capacity during high‐intensity exercise compared with typically developing individuals. However, it is important to interpret this finding with caution, as this measure may underestimate true breathing capacity in populations with severe neuromuscular diseases.^37^

Additionally, low tidal volumes and an elevated breathing frequency during exercise increase dead space volumes, thereby reducing ventilatory efficiency in the lungs as dead space makes up a larger proportion of each breath. Moreover, an increased dead space volume has also been suggested to influence measurements of respiratory exchange ratio. Specifically, in a situation with increased dead space, an elevated respiratory exchange ratio might be observed without a concomitant shift in substrate utilization, making our findings of a similar development of respiratory exchange ratio over time during exercise less clear to interpret in participants with CP.^38^ Increased work of breathing also increases the demand for oxygen in the respiratory muscles, potentially leading to premature fatigue of the diaphragm.^39^ The increased amount of blood redirected towards the diaphragm and accessory respiratory muscles results in a reduced blood flow to actively working muscles and thereby a reduced functional capacity.^40^

Altogether, these alterations and phenomena could potentially contribute to exercise limitations in individuals with CP, as supported by our findings of a negative drop in the EMG Fatigue Index in the vastus lateralis and gastrocnemius medialis muscles during the incremental exercise test to task failure.

Certain considerations must be taken into account when interpreting the findings of this study. Both incremental tests used in this study are novel for individuals with CP. However, we have recently shown that the incremental exercise test to task failure used in the current investigation is valid and feasible in this population.^41^ Additionally, the test protocols were individualized by experts in cardiopulmonary exercise testing, incorporating both subjective and objective criteria to ensure maximal performance. However, the evaluation of respiratory frequency as a proportion of cardiovascular intensity is a novel approach, and its interpretability may require further exploration. In addition, maximum heart rate in individuals with CP has in some reports been described to be lower than in typically developing individuals;^4^, ^23^ however, contradictory data have also been presented.^20^, ^42^ Thus, the use of the formula 220 − age to estimate HR_max_ might need further validation in individuals with CP. Furthermore, most of our study participants were classified in GMFCS levels III to IV and had previous familiarity with frame running. In addition, they all maintained a moderate level of physical activity on a regular basis. These factors may limit the generalizability of our results to all individuals with CP. Owing to the limited sample size, we have also refrained from analysing our data on the basis of GMFCS level. It should also be noted that, for the participants with CP, the dynamic spirometry was performed 1 year after the initial data collection, and it is possible that lung function could have altered during this time frame. Similarly, ECG data were also collected 1 year after the initial data collection for all participants (typically developing and CP). Lastly, the raw data obtained during the dynamic spirometry from individuals with CP might be slightly underestimated compared with typically developing individuals owing to the use of a face mask instead of the traditional mouthpiece.^43^

In conclusion, our study demonstrates a disproportional ventilatory response in individuals with CP during incremental exercise, where metabolic perturbations do not seem to be the driving factor behind the altered respiratory response. Instead, the increased respiratory frequency is linked to an inability to increase breath volume during incremental exercise, which in turn emerges as a potential contributor to the observed onset of fatigue. Our findings emphasize the need for further investigations aimed at elucidating the specific factors driving the altered respiratory response in individuals with CP. Additionally, exploring interventions targeted at improving ventilatory function in individuals with CP is warranted, potentially offering insights into optimizing their exercise capacity and overall health.

## CONFLICT OF INTEREST STATEMENT

The authors have stated that they had no interests which might be perceived as posing a conflict or bias.

## FUNDING INFORMATION

Norrbacka‐Eugenia Stiftelsen, Stiftelsen Sunnerdahls Handikappfond, The Elsass Foundation, Stiftelsen Promobilia, Phelps Stichting voor Spastici, JKF Kinderfonds, Kinderrevalidatie Fonds Adriaanstichting.

## Supporting information


**Figure S1:** Ventilatory efficiency during the submaximal incremental test in individuals with cerebral palsy and typically developing participants.

**Table S1:** Physiological response during incremental exercise test to task failure.

**Table S2:** Statistical summary.

## Data Availability

The data that support the findings of the study are available as downloadable files from: Figshare.com; https://doi.org/10.6084/m9.figshare.30998299.
