# Supplementary figures and images for: Disproportional ventilatory response to incremental exercise in individuals with cerebral palsy
Source: Dev Med Child Neurol. 2026 Jan 18;68(8):1139–51. doi: 10.1111/dmcn.70164 (PMC13340625; doi:10.1111/dmcn.70164)

# Ventilatory Efficiency

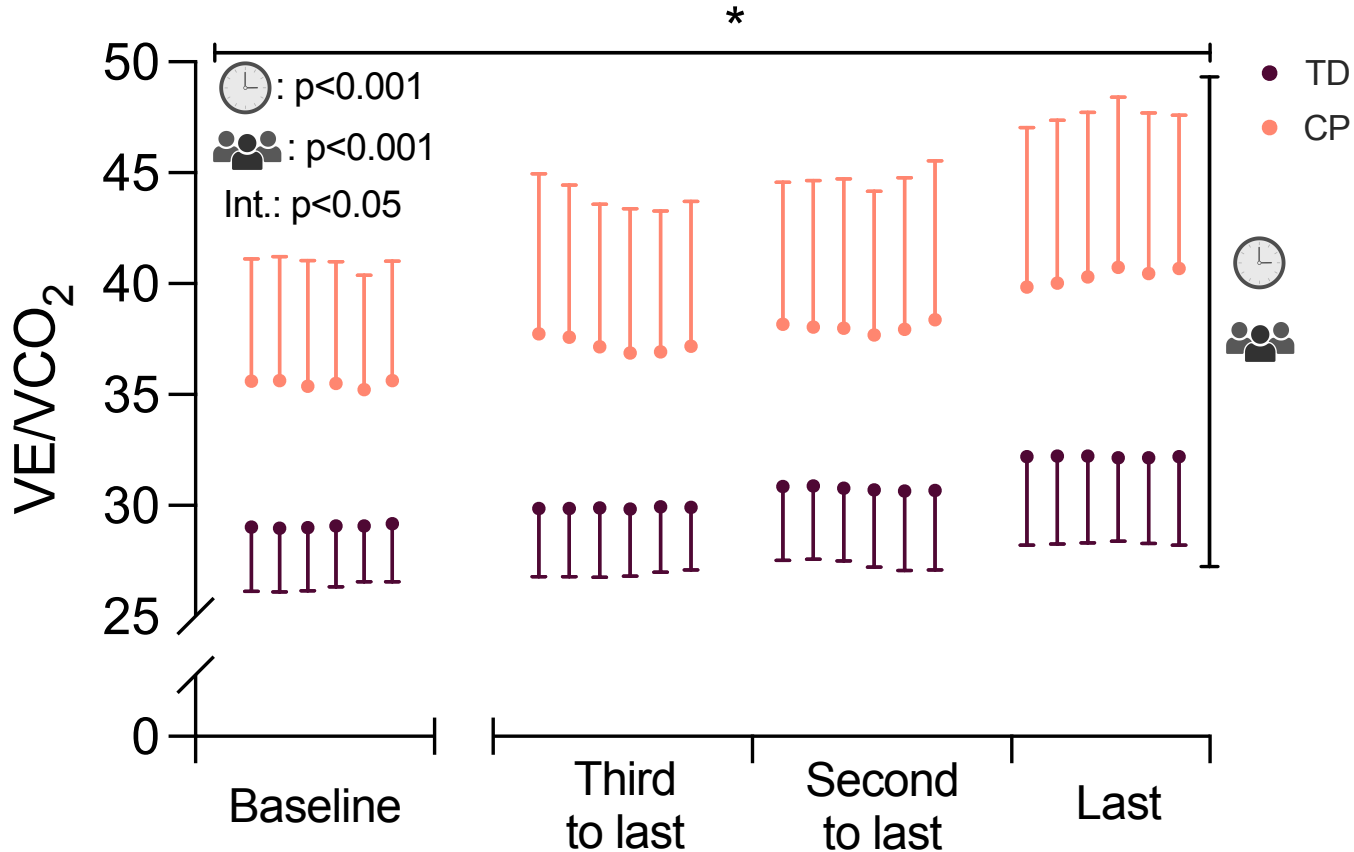

Supplement: Supplementary file 1 — Figure S1: Ventilatory efficiency during the submaximal incremental test in individuals with cerebral palsy and typically developing participants. [file DMCN-68-1139-s003.pdf]
