# Supplementary material for: Disproportional ventilatory response to incremental exercise in individuals with cerebral palsy
Source: Dev Med Child Neurol. 2026 Jan 18;68(8):1139–51. doi: 10.1111/dmcn.70164 (PMC13340625; doi:10.1111/dmcn.70164)
Supplement: Supplementary file 2 — Table S1: Physiological response during incremental exercise test to task failure. [file DMCN-68-1139-s002.docx]

| **TABLE S1** Physiological response during incremental exercise test to task failure | | | | | |
| --- | --- | --- | --- | --- | --- |
|  | **HR_peak_** | **RER_peak_** | **Lactate** | **RPE** | **Signs of perceived exertion** |
|  | *<18 years; HR ≥ 185 bpm^(1)^*  *≥18 years; HR ≥ 85% of the age-predicted (220-age)^(2)^* | *RER ≥1.0^(1)^* | *≥ 6 mmol/L^(1)^* | *≥ 17^(1)^* | *Out of breath, sweating, fatigued and/or unable to continue the test^(1)^* |
| **Typically developed** | |  |  |  |  |
| **1** | 202 | 1,10 | 11,1 | - | ✓ |
| **2** | 196 | 1,24 | 11,5 | - | ✓ |
| **3** | 173 | 0,93* | 4,4* | 17,5 | ✓ |
| **4** | 192 | 1,08 | 13,1 | 19 | ✓ |
| **5** | 216 | 1,01 | 11,3 | 17 | ✓ |
| **6** | - | 1,19 | 7,2 | 16,5* | ✓ |
| **8** | 199 | 1,27 | 14,2 | 20 | ✓ |
| **9** | - | 1,04 | 8,4 | 18 | ✓ |
| **11** | 203 | 1,11 | 9,4 | 19,5 | ✓ |
| **12** | 200 | 1,03 | 7,8 | 18,5 | ✓ |
| **13** | 208 | 1,02 | 6,8 | 20 | ✓ |
| **14** | 199 | 1,13 | 10,9 | 19 | ✓ |
| **15** | 202 | 1,03 | 9,2 | 19 | ✓ |
| **16** | 197 | 0,93* | 8,6 | 18,5 | ✓ |
| **17** | 203 | 1,01 | 6,9 | 20 | ✓ |
| **18** | 210 | 1,02 | 10,2 | 19,5 | ✓ |
| **19** | 218 | 1,09 | 11,9 | 19 | ✓ |
| **20** | 185 | 0,98* | 8,3 | 19 | ✓ |
| **21** | 195 | 1,05 | 12,3 | 19,5 | ✓ |
| **22** | 189 | 1,06 | 12,2 | 19 | ✓ |
| **23** | 207 | 1,06 | 11,5 | 20 | ✓ |
| **24** | 202 | 1,10 | 8,0 | 20 | ✓ |
| **25** | 204 | 1,06 | 12 | 20 | ✓ |
| **26** | 193 | 1,09 | 13,4 | 18 | ✓ |
| **27** | 200 | 1,01 | 10,2 | 19,5 | ✓ |
| **29** | 193 | 1,01 | 11,5 | 16,5* | ✓ |
| **30** | 199 | 1,15 | 9,8 | 19,5 | ✓ |
| **61** | 216 | 1,18 | 10,9 | 20 | ✓ |
| **62** | 182 | 1,00 | 9,4 | 20 | ✓ |
| **63** | 185 | 1,12 | 16,1 | 19 | ✓ |
| **Cerebral palsy** | |  |  |  |  |
| **31** | 151* | - | 3,3* | 19 | -* |
| **32** | 203 | 1,05 | 13,6 | 19,5 | ✓ |
| **33** | 188 | 0,99* | 9,0 | 19 | ✓ |
| **34** | 185 | 1,11 | 5,9* | 20 | ✓ |
| **36** | 192 | 1,08 | 5,1* | 20 | ✓ |
| **37** | 188 | 0,91* | 3,3* | 18,5 | ✓ |
| **38** | 200 | 0,94* | 3,4* | 20 | ✓ |
| **39** | 168 | 0,94* | 2,7* | 15* | ✓ |
| **40** | 183 | 1,19 | 5,3* | 19 | ✓ |
| **41** | 187 | 1,20 | 9,9 | 16* | ✓ |
| **43** | 171 | 1,04 | 8,9 | 19 | ✓ |
| **44** | 193 | 1,00 | 3,6* | 18 | ✓ |
| **45** | 180 | 0,86* | 4,6* | 18,5 | ✓ |
| **46** | 174 | 1,22 | 12,0 | 18,5 | ✓ |

* Denotes not fulfilled criteria. Abbreviations: HR_peak_; Heart Rate peak: RER_peak_; Respiratory Exchange Ratio peak: RPE; Rate of Perceived Exertion

1. Falk B, Dotan R. Measurement and Interpretation of Maximal Aerobic Power in Children. Pediatr Exerc Sci. 2019;31(2):144-51.

2. Medicine ACoS. ACSM's guidelines for exercise testing and prescription. Tenth edition ed. Philadelphia: Wolters Kluwer; 2018.
